# Supplementary material for: A UMLS-based spell checker for natural language processing in vaccine safety
Source: BMC Med Inform Decis Mak. 2007 Feb 12;7:3. doi: 10.1186/1472-6947-7-3 (PMC1805499; doi:10.1186/1472-6947-7-3)
Supplement: Additional file 13 — RAP application directory. Sets up directory for RAP (RDF application for PHP) source code [file 1472-6947-7-3-S13.gz › rap/doc/index.html.bak]

RAP - Rdf API for PHP V0.9.1 - Home


RDF API Home |
Documentation |
PHPDoc

# RAP - RDF API for PHP V0.9.2

RAP is a software package for parsing, searching, manipulating, serializing
and serving RDF models.

Its features include:

- statement-centric methods
  for manipulating an RDF model as a set of RDF triples- resource-centric methods
    for manipulating an RDF model as a set of resources- ontology-centric methods
      for manipulating an RDF model through vocabulary specific methods- quad- and named graph-centric methods for manipulating RDF datasets- integrated RDF/XML, N3, N-TRIPLE, TriX and GRDDL parser- integrated RDF/XML, N3, N-TRIPLE, TriX serializer- in-memory or database model storage- support for the RDQL query language- inference engine supporting RDF-Schema reasoning and some OWL entailments
                - integrated RDF server providing similar functionality as the Joseki
                  RDF server
                - graphical user-interface for managing
                  database-backed RDF models
                - support for common vocabularies
                - drawing graph visualizations

For further information about RAP see:

- Introduction to RAP.- Documentation for tutorials, usage examples,
    and implementation notes.- RAP PHPDoc for the detailed API documentation
      covering all classes and methods.- RAP Mailing List- Interactive API
          demonstration to test the API with your own RDF data.- Interactive
            RDQL demonstration to test the RDQL engine with your own RDF data
            and queries.- RAP RDQL grammar and RAP
              RDQL Test Cases.- RDF server installation and usage manual

---

### Download and Installation

1. Download the current version from: http://sourceforge.net/projects/rdfapi-php/- Unpack the zip file.- Include RDF API into your scripts:
       - define("RDFAPI\_INCLUDE\_DIR", "C:/Apache/htdocs/rdf\_api/api/");- include(RDFAPI\_INCLUDE\_DIR . "RDFAPI.php");
       - Change the constant RDFAPI\_INCLUDE\_DIR to the directory in which you
         have unpacked the zip file.

RAP can be used with PHP 4 or PHP 5.

- When used with PHP 4 it requires at least version 4.2- When used with PHP 5 it requires at least version 5.0.1

---

### License

- RAP can be used under the terms of the GNU
  LESSER GENERAL PUBLIC LICENSE (LGPL).

---

### Status of the code

| Version | Comment | Release Date |
| --- | --- | --- |
| V0.9.2 | Support for Named Graphs and RDF dataset added.  Graph visualization modul added.   Multiple bugs fixed. | 2005-07-06 |
| V0.9.1 | GRDDL parser added.  Improved RDF/XML namespace handling.  Option to create models using RDQL added.   RDF DB Utils bugs fixed.  Improved common vocabulary support.   RSS, FOAF, ATOM vocabularies added. | 2004-12-10 |
| V0.9 | Resource-centric ResModel API added.  Ontology-centric OntModel API added.  InfModelF and InfModelB inference engine added.   RDF DB Utils added.  RAP Unit Test Suite extended. | 2004-10-21 |
| V0.8.1 | New MemModel indexing speeding up searches.  Dynamic script inclusion speeding up many operations.  New FindIterator() class added.  RAP Unit Test Suite added.  Multiple bugs fixed. | 2004-08-24 |
| V0.8 | RDF server supporting the RDF NetAPI added.  RDQL syntax updated according to the W3C member submission.  Multiple bug fixes. | 2004-06-01 |
| V0.7.1 | Multiple bugs fixed.   Support for XML default namespace (xmlns="uri\_namespace"). | 2004-03-29 |
| V0.7 | Improved RDF, N3 and N-Triple parsers and serializers.  Support for the "RDF/XML Syntax Specification (Revised) -   W3C Working Draft 10 October 2003".  RDQLResultIterator added. | 2003-11-19 |
| V0.6 | Database backend, rdql and n3 support added. | 2003-07-31 |
| V0.5 | Parser and model operations are much faster now.   Search index and some convenience methods added. | 2003-06-01 |
| V0.4 | Support for common vocabularies and StatementIterator object added. | 2003-02-20 |
| V0.3 | Support for rdf:datatype and rdf:nodeID added. | 2003-01-13 |
| V0.2 | Some bugs fixed.  Documentation updated. | 2002-10-08 |
| V0.1 | Initial Release. | 2002-09-24 |

---

### Contributions

RAP is an open source project and we highly welcome any kind of useful
external contributions.

### Lots of thanks to

- Daniel Westphal for the ResModel- and OntModel-APIs, RDF dataset and the RDF-S/OWL
  reasoning support.
- Radoslaw Oldakowski for the database backend and the RDQL query engine
  and to
- Gunnar AA. Grimnes for the N3 support and the RDF DB Utils package
- Phil Dawes for the NetAPI implementation
- Tobias Gauß for the MemModel indexing and the unit test suite.
- Anton Köstlbacher for the graph visualization.
- Lukas Smith, Sören Auer, Leandro Mariano Lopez, Riccardo Catanzani,
  Scott Willy for multiple bug reports and fixes.

### Open issues

There are some open issues and future ideas which await implementation.  
So if you want to contribute some code to RAP, our Open
Issues list might give you some valuable ideas :-)

---

### Other RDF tools for PHP

- Section on PHP in
  Developers Guide to Semantic Web Toolkits for different Programming Languages- pOWL (Sören
    Auer): Webbased OWL editor and OWL API based on RAP.- appmosphere RDF
      classes (Benjamin Nowack): Lightweight RDF parser and N-Triples
      serializer.- SimpleRdfParser
        (Morten Frederiksen): A wrapper class around the RAP rdf parser, which
        parses RDF into a simple array. The SimpleRdfParser is faster than the
        RAP parser because it doesn't use the rest of the RAP library.- Rdfworld.php (Chris
          Snyder): Rdfworld.php is an RDF to PHP object converter, inspired by
          Aaron Swartz's TRAMP for Python.  
          It allows PHP developers to convert RDF Resources into PHP objects with
          appropriate properties. The converter is based on RAP V0.3.- PHP XML Classes
            (Luis Argerich): XML/RDF package including RDF parser, storage mechanism
            and RDQL query interface. The RAP rdf parser is an up-to-date version
            of the rdf parser from this package. Luis' parser and RDQL query engine
            haven't been changed for 2 years and don't reflect the current RDF specification
            any more.- rdftp (Vangelis Vassiliadis):
              rdftp is an RDF server implemented as a PHP script that supports Query
              and Update operations on RDF content.

---

### References

- Further RAP usage examples are found in:

- Exposing
  Web application data semantically using RAP php|architect, issue
  October-2004
- Practical
  RDF by Shelley Powers - O'Reilly book: Practical RDF, Chapter
  9
- Parsing
  FOAF with PHP by Ian Davis

- W3C: RDF Core Test Results Comparison for different RDF
  APIs- PHP: RDF Parser by Luis Argerich - W3C: Resource Description
      Framework (RDF)
    - Workshop on Scripting for the Semantic Web at ESWC 2005
    - For a genaral overview about other RDF resources and tools see: Dave
      Beckett's Resource Description Framework (RDF) Resource Guide

---

### Feedback

Please send:

+ general questions about RAP  
+ feature requests  
+ bug reports  
+ patches  
+ links to interesting RAP use cases

to rdfapi-php-interest@lists.sourceforge.net

You can subscribe rdfapi-php-interest and scroll through the mailing list archives under http://lists.sourceforge.net/lists/listinfo/rdfapi-php-interest

Other feedback can also be send directly to: chris@bizer.de (Chris Bizer)

---

RDF API Home |
Documentation |
PHPDoc
